# Prediction of femoral strength using 3D finite element models reconstructed from DXA images: validation against experiments

**DOI:** 10.1007/s10237-016-0866-2

**Published:** 2016-12-21

**Authors:** Lorenzo Grassi, Sami P. Väänänen, Matti Ristinmaa, Jukka S. Jurvelin, Hanna Isaksson

**Affiliations:** 1 Department of Biomedical Engineering, Lund University, BMC D13, 221 84 Lund, Sweden; 2 Department of Applied Physics, University of Eastern Finland, Kuopio, Finland; 3 Department of Orthopaedics, Traumatology and Hand Surgery, Kuopio University Hospital, Kuopio, Finland; 4 Division of Solid Mechanics, Lund University, Lund, Sweden; 5 Diagnostic Imaging Center, Kuopio University Hospital, Kuopio, Finland

**Keywords:** Statistical shape model, Statistical appearance model, Finite element, Proximal femur, Validation

## Abstract

Computed tomography (CT)-based finite element (FE) models may improve the current osteoporosis diagnostics and prediction of fracture risk by providing an estimate for femoral strength. However, the need for a CT scan, as opposed to the conventional use of dual-energy X-ray absorptiometry (DXA) for osteoporosis diagnostics, is considered a major obstacle. The 3D shape and bone mineral density (BMD) distribution of a femur can be reconstructed using a statistical shape and appearance model (SSAM) and the DXA image of the femur. Then, the reconstructed shape and BMD could be used to build FE models to predict bone strength. Since high accuracy is needed in all steps of the analysis, this study aimed at evaluating the ability of a 3D FE model built from one 2D DXA image to predict the strains and fracture load of human femora. Three cadaver femora were retrieved, for which experimental measurements from ex vivo mechanical tests were available. FE models were built using the SSAM-based reconstructions: using only the SSAM-reconstructed shape, only the SSAM-reconstructed BMD distribution, and the full SSAM-based reconstruction (including both shape and BMD distribution). When compared with experimental data, the SSAM-based models predicted accurately principal strains (coefficient of determination >0.83, normalized root-mean-square error <16%) and femoral strength (standard error of the estimate 1215 N). These results were only slightly inferior to those obtained with CT-based FE models, but with the considerable advantage of the models being built from DXA images. In summary, the results support the feasibility of SSAM-based models as a practical tool to introduce FE-based bone strength estimation in the current fracture risk diagnostics.

## Introduction

Fragility fractures represent a major concern in the modern Western society, with both fracture incidence and associated economic burden continuously increasing (Burge et al. 2007). The majority of low-energy trauma fractures can be ascribed to bone weakness due to osteoporosis (Johnell and Kanis 2006). While pharmacological treatments can increase the strength of osteoporotic bone and reduce the risk of fracture (Kanis et al. 2013), the identification of the subjects at high risk of fracture remains an issue. The methods currently adopted in the clinical practice are based on the measurement of bone mineral density (BMD) using dual-energy X-ray absorptiometry (DXA), often complemented by epidemiological and statistical parameters (Kanis et al. 2005; Cummings et al. 2006). These methods are limited in their ability to accurately diagnose osteoporosis [30% false negatives (Järvinen et al. 2005, 2014)], with the epidemiological and statistical tools often not being general enough, due to their ethnic specificity (Watts et al. 2009; Lekamwasam 2010; Silverman and Calderon 2010).

Subject-specific finite element (FE) models have the potential to improve the accuracy of fracture risk predictions by providing an accurate estimate for bone strength, together with a comprehensive and local characterization of the mechanical response of bone under different loading conditions. Although FE models can predict femoral strength more closely, as compared to BMD based on DXA images (Cody et al. 1999), they are still not used in the clinics to predict fracture risk. One reason for this is that the majority of the proposed FE modelling techniques is based on computed tomography (CT) datasets. When compared with DXA, CT has higher operational cost and provides a higher radiation dose to the patients (Kanis 2002; Engelke et al. 2015). Subject-specific FE models from DXA images would overcome this issue, enabling also the possibility of conducting clinical trials in parallel with the current diagnostics. When building FE models from DXA images, the two main approaches are: (1) construction of two-dimensional FE models using the planar image provided by the DXA instrument (Op Den Buijs and Dragomir-Daescu 2010; MacNeil et al. 2012; Sarkalkan et al. 2014a; Yang et al. 2014; Dall’Ara et al. 2016), and (2) use of statistical tools (most often based on principal component analysis, PCA) to reconstruct the 3D shape and BMD distribution from a planar DXA image and use the reconstructed information to perform a 3D FE analysis (Langton et al. 2009; Whitmarsh et al. 2011; Väänänen et al. 2015). Two-dimensional FE models based on DXA may accurately predict femoral strength (Yang et al. 2014; Dall’Ara et al. 2016), but cannot overcome the limitations inherent to their two-dimensional nature, such as the inability to test the bone in out-of-plane direction or to localize the point where the fracture originates. The 3D shape and BMD reconstruction from a 2D image using statistical tools has the potential to overcome these issues. The accuracy in the reconstruction of both shape and BMD has reached remarkable levels [average shape reconstruction error 1.4 mm, mean absolute difference of the reconstructed volumetric BMD $185\,\hbox {mg/cm}^{3}$ (Väänänen et al. 2015)].

**Table 1:** Patient information (sex, age at death, height, weight, BMD at femoral neck, and leg side) for the three samples used in this study

Specimen ID	Sex (M/F)	Age (years)	Height (cm)	Weight (kg)	Neck BMD ($\hbox {g/cm}^{3}$)	Side (L/R)
#1	M	22	186	106	1.16	L
#2	M	58	183	85	0.6	R
#3	M	58	183	112	0.89	L

However, to the authors’ best knowledge, no 3D FE models obtained from statistical reconstruction of a DXA image have been confirmed to accurately predict the mechanical behaviour of human femora, and ultimately, the bone strength (Sarkalkan et al. 2014b; Castro-Mateos et al. 2014). Bryan et al. (2009) used a statistical model to generate 1000 realistic femur anatomies and estimate their fracture risk in a configuration resembling a postero-lateral fall. However, the generated models used material properties from CT data, and no direct validation could be provided, since the models were randomly generated. Whitmarsh et al. (2012) used a statistical reconstruction of shape and BMD from DXA images to discriminate hip fracture cases. The contribution of the reconstructed models was restricted to the extraction of three-dimensional anatomical shape and density parameters. These were used as additional risk factors to improve the accuracy of the discrimination. Thus, no actual FE analyses of the mechanical behaviour of the reconstructed models were performed. Grassi et al. (2014a) evaluated the ability of PCA-based finite element models to predict the mechanical behaviour of 8 human femora. A high correlation was found between the strains predicted by the reconstructed PCA-based models and those measured during analogous experimental tests on the same specimens. However, the PCA-based models were reconstructed against 3D CT data, and no validation of femoral strength was provided. Thevenot et al. (2014) proposed a specific method to construct 3D FE models of proximal femora from a single radiograph, using a shape template and a set of geometrical parameters that were measured from the radiograph. The models were used to predict femoral strength on 21 samples in a condition resembling a fall to the side, showing a promising accuracy (coefficient of determination $=$ 0.64, standard error of the estimate $=$ 543 N). The material properties for the models were estimated based on the CT-based values of the training set bones and a homogeneity index derived from the radiograph. Therefore, the subject-specific BMD distribution was not taken into account, which can be a limitation when samples with BMD significantly different from that of the seven bones of the training set are examined. Recently, Bonaretti et al. (2014) created statistical models of shape and appearance using both an image-based approach (i.e. the result of the reconstruction is a volumetric image) and a mesh-based approach (i.e. a FE-ready mesh is reconstructed and used to store the shape and appearance information in the statistical model), and their strain predictions were compared to those of FE models built from segmentation of the original CT images. Both image-based and mesh-based approaches predicted similar principal strains when compared to the CT-based models, but with the mesh-based approach being more compact (i.e. requiring less modes of variation to provide an accurate reconstruction) and significantly less computationally intensive. The study concluded that image-based approaches were preferred, since some severely distorted elements were found when using the mesh-based approach. However, element distortion can be mitigated by using a mesh relaxation algorithm and by implementing a modified cost function for bone reconstruction (Väänänen et al. 2015).

Recently, our group presented a mesh-based statistical shape and appearance model (SSAM) to reconstruct shape and BMD of a proximal femur from a single DXA image (Väänänen et al. 2015), as well as a subject-specific FE modelling procedure from CT scans to predict strain and strength of human proximal femora (Grassi et al. 2016). The latter study was validated against a set of full-field experimental measurements collected using digital image correlation (DIC) (Grassi et al. 2014b). In the present study, subsequently, we aimed at evaluating the ability of a SSAM-based FE model to accurately predict strains and strength in human femora. The results were validated against experimental DIC data and compared to the performance of analogous CT-based FE model.

## Material and methods

### Materials

Three male cadaver human femora, harvested fresh at Kuopio University Hospital, Finland (ethical permission 5783/2004/044/07), were used for this study. Height, weight, sex, BMD at the femoral neck, and age at death are presented in Table 1. None of the donors had any reported musculoskeletal disease. The specimens were scanned both with CT (Somatom Definition AS64, Siemens AG, $0.4 \times 0.4 \times 0.6$ mm voxel size) and with two DXA devices (Lunar Prodigy and Lunar iDXA, GE Healthcare, pixel size $1.05 \times 0.60$ mm and $0.25 \times 0.3$ mm, respectively). For all specimens, experimental strain measurements were obtained from mechanical tests performed up to fracture in a configuration resembling single leg stance. The force versus displacement curves were acquired from the loading device, while the full-field strain distribution was acquired using DIC (Grassi et al. 2014b).

### Creation of the models

The SSAM has been thoroughly described earlier (Väänänen et al. 2015) and is only briefly summarized here. A training set of 34 proximal femur anatomies was retrieved (Finnish population, 13 right and 21 left, 30 men and 4 women, age $50 \pm 16$ years old, age range 18–82). The samples were segmented, and their average shape was calculated. A template mesh of the average shape was generated (1.6 million tetrahedral elements, Hypermesh 11.0, Altair Engineering, Inc.) and morphed over the shape of each bone in the training set. A MATLAB (The Mathworks, Inc.) re-implementation of Bonemat_V2 (Taddei et al. 2007; Venäläinen et al. 2016) was used to map bone density (as obtained via calibration of the CT images using a dipotassium phosphate phantom, model 3CT, Mindways, Inc.) over each morphed mesh based on the underlying calibrated CT values. The SSAM was created by performing the singular value decomposition of a matrix containing the nodal coordinates of each morphed tetrahedral mesh and the density values for each element, arranged columnwise. The reconstruction of a femur from its 2D image was performed by using a genetic algorithm to register the SSAM to the 2D reference image. A digital reconstructed radiography (DRR) was generated at each iteration round by projecting the SSAM instance onto the coronal plane. The cost function of the genetic algorithm was given by the sum of three components: the sum of absolute difference of the areal BMD between DRR and the 2D reference image, the mesh quality of the instance (Liu and Joe 1994), and the anatomical positioning. For each of the present samples, the reconstruction was performed using three different 2D reference images, namely 2D projection of the CT image along the antero-posterior plane (hereafter referred to as CTproj, created to represent the optimum in terms of signal-to-noise ratio), the DXA image obtained with Lunar Prodigy (lower resolution, hereafter referred to as Prodigy), and the DXA image obtained with Lunar iDXA (higher resolution, hereafter referred to as iDXA).

The CT-based FE modelling procedure has also been previously described in detail (Grassi et al. 2016). Briefly, the femur geometry was retrieved through semi-automatic segmentation of the CT images. The geometry was converted to non-uniform rational B-splines and meshed ($\sim$100 k elements, Hypermesh v13.0). Inhomogeneous isotropic Young’s moduli were assigned using Bonemat_V3 (Taddei et al. 2007): first, the calibrated Hounsfield units were converted to equivalent radiological density of dipotassium phosphate using a calibration phantom (model 3CT, Mindways, Inc.). Next, a set of empirical relationships linked the equivalent radiological density to the modulus of elasticity (Schileo et al. 2008), and the modulus for each finite element was obtained by numerical integration over the element volume.

The CT-based FE modelling procedure was combined with the SSAM and the reconstruction algorithm to build subject-specific FE models from a single DXA image. For each sample, three different reference images were used for reconstruction (CTproj, iDXA, and Prodigy). Three different models were built for each of the three samples (#1, #2, and #3), and for each of the three reference images used for reconstruction:

1. *SSAM–BMD* models: these models were obtained using the CT-based geometry (considered as the true bone shape) and the bone density as estimated from the SSAM-based reconstruction of the DXA image.
2. *SSAM-shape* models: obtained using the estimated bone geometry as reconstructed by registering the SSAM on the DXA image and the bone density from calibrated CT values (considered as the true bone density distribution).
3. *SSAM-shape* and *BMD* models: these models were obtained using both the estimated geometry and the estimated bone density as reconstructed by registering the SSAM on the DXA image.

The rationale behind these three models was to evaluate the individual effects of each step in the shape (*SSAM-shape* models) and BMD reconstruction (*SSAM–BMD* models) on the final accuracy obtained by models implementing both shape and BMD as reconstructed by registering the SSAM on the DXA image (*SSAM-shape* and *BMD* models).

The *SSAM–BMD* models were created as follows: the CT-based FE meshes used in Grassi et al. (2016) were retrieved, and the bone density distribution was mapped based on the reconstructed BMD obtained by registration of the SSAM on the DXA image. Therefore, the model obtained by registering the SSAM on the DXA image (hereafter referred to as SSAM-based mesh) was first registered and then morphed to the CT-based geometry. The BMD in the SSAM-based mesh was presented as a three-dimensional step function according to the element borders. Then, the BMD was captured into the target CT-based mesh by integrating the function over each element in the target mesh. As a result, the density at each element was given by the average of the densities in the SSAM-based mesh, weighted by the volume of intersection between the element itself with each element of the SSAM-based mesh. Young’s moduli were retrieved from density values using the same density–elasticity relationship as adopted for CT-based FE models. After the mapping, a two-step compensation process was applied, where: (1) the modulus of elasticity of the surface elements was derived as the maximum between the mapped value and the moduli of the neighbouring elements that were not surface elements as well, and (2) the allowed maximum modulus of elasticity for the model was set to 22 GPa (Bayraktar et al. 2004), while the minimum modulus of elasticity for the surface elements was set to 5 GPa [assuming very thin cortex and consequently a Young’s modulus corresponding to that of the underlying trabecular bone (Rho et al. 1993)]. The whole registration, warping, and density mapping procedure were implemented in MATLAB.

**Fig. 1:**
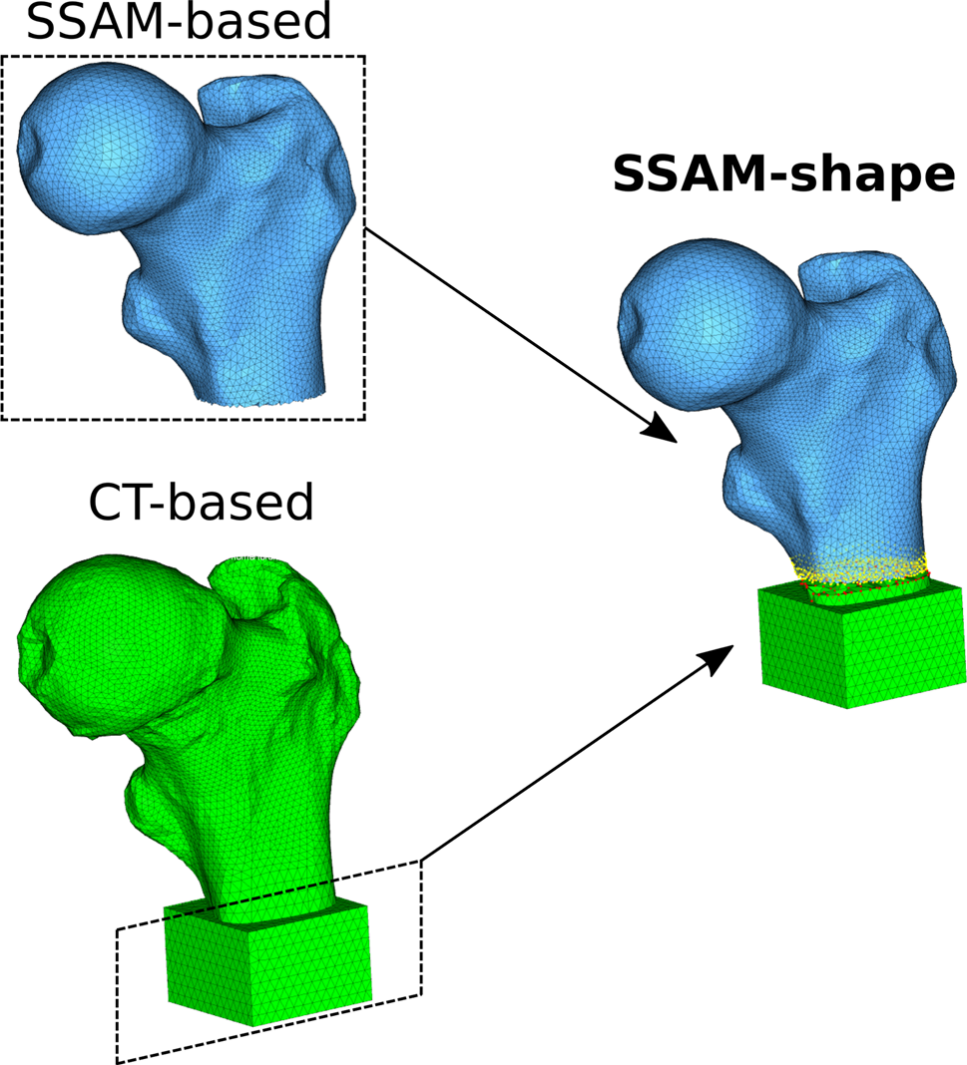
Schematic of the generation of the FE models implementing the SSAM-based shape (*SSAM-shape* and *SSAM-shape* and *BMD* models): the model produced by the SSAM-based reconstruction (depicted in *blue*, *left side*) presents a shorter shaft than the actual sample, as reconstructed by segmentation of the its CT scan (CT-based model depicted in *green*, *left side*). In order to test the SSAM-shape-based models while keeping the exact same boundary conditions as in the experiments (Grassi et al. 2014a, b) and in the CT-based FE models (Grassi et al. 2016), the most distal part of the CT-based FE model was added to the SSAM-based FE model and connected to it using tie constraints (Abaqus v2016, Dassault Systèmes). The distal cut region of the SSAM-based FE model (*yellow points*) was thus rigidly connected to the cutting region of the CT-based FE model (*red points*)

The *SSAM-shape* models were created by taking the geometry of the SSAM-based mesh. The geometry was meshed using Hypermesh (v14.0, Altair, Inc.), using the same parameters adopted in Grassi et al. (2016) (element size 1.5 mm on the femoral neck, 2 mm elsewhere, $\sim$100 k tetrahedral elements). The mesh was then registered to the CT reference system, and the bone density values were assigned based on the underlying CT values using Bonemat_V3 (Taddei et al. 2007). The geometry of the SSAM-based mesh included a smaller portion of the bone than the femoral segment imaged with CT. In order to create *SSAM-shape* models with the same length as that of the CT-based models, the missing distal part of the shaft and the epoxy pot from the CT-based models were connected to the model using tie connections in Abaqus (v2016, Dassault Systèmes). These procedures were implemented in MATLAB. An example of the model is shown in Fig. 1.

The *SSAM-shape* and *BMD* models were created by taking the shape of the reconstructed SSAM-based models, analogously to what described for the *SSAM-shape* models, as well as including the bone density from the reconstructed SSAM-based models, using the procedure described for the *SSAM–BMD* models.

### Performance comparison

In order to evaluate the performance of each of the models created, identical boundary conditions to those in the experiments (Grassi et al. 2014b) and in the CT-based FE models (Grassi et al. 2016) were applied. The ability of the models to predict the mechanical behaviour of bone was then evaluated both in terms of strain prediction accuracy and of ability to predict femoral strength.

To assess the strain prediction accuracy, a force equal to four times the body weight (BW) of the subject was applied onto the femoral head, equally distributed among the 10 most superior nodes on the surface. The principal strain patterns were then obtained and compared to principal strains measured experimentally with DIC. To do this, the DIC cloud was registered over the FE model using an iterative closest point algorithm. When the model had its shape retrieved from SSAM reconstruction (*SSAM-shape* and *SSAM-shape* & *BMD* models), a point-to-surface projection of the DIC points over the FE model was performed. For each surface element of the FE models, the smallest sphere circumscribing it was calculated. All DIC data within that sphere were averaged, and the obtained experimental value was compared to the FE element strain. A robust regression analysis with bi-square weighting function of the major and minor principal strain magnitudes was finally performed. The coefficient of determination, slope, intercept, normalized root- mean-square error (NRMSE) and maximum error were reported for each robust regression. The same accuracy parameters obtained earlier by the CT-based FE models (Grassi et al. 2016) are also reported to allow for a comparison between the proposed SSAM-based models and the state of the art.

The error in the shape reconstruction was also assessed. The distance between the nodes of the *SSAM-shape* models and the surface of the CT-based models was calculated. In addition, the volumetric difference between the *SSAM-shape* and CT-based models was calculated, limited to the femoral neck region.

A robust regression analysis of the experimental versus predicted principal strains was also performed considering only the femoral neck region, for all models and reference images, and the accuracy parameters are compared to those obtained by CT-based FE models in the same anatomical region (data retrieved and processed from Grassi et al. 2016).

**Fig. 2:**
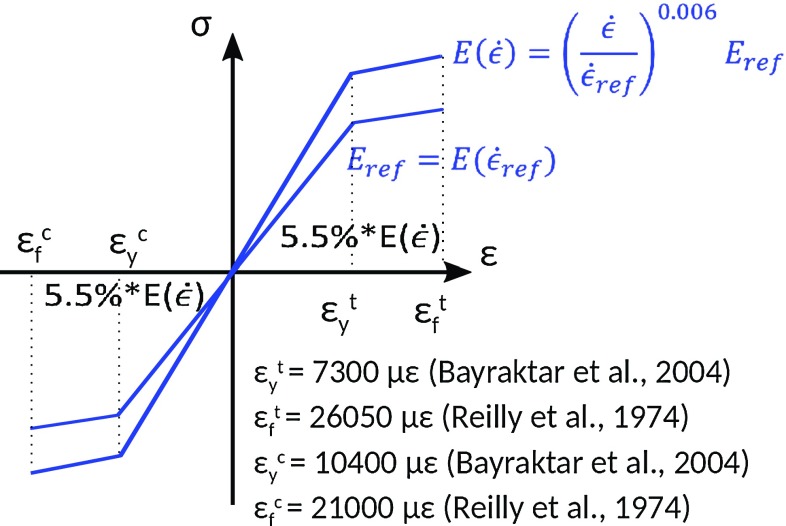
Diagram showing the material model implemented to predict femoral strength, as proposed first in Grassi et al. (2016). Each element is assigned a modulus of elasticity which applies for the reference strain rate [5000 $\upmu \upvarepsilon$/s, consistently with the strain rate used to experimentally obtain the density–elasticity relationships (Morgan et al. 2003) and yield limit values (Bayraktar et al. 2004) used in this model]. The strain rate was then constantly updated for each element during the simulation and its modulus of elasticity according to relationship for $E({\dot{\epsilon }})$ shown in figure. Yield and failure were defined by separate thresholds for tension and compression. When an element reached the yield state, its modulus of elasticity was reduced to $0.55*E({\dot{\epsilon }})$, and the simulation proceeded. The simulation was stopped when the first surface element reached the failed state, and the applied force at that stage taken as the predicted femoral strength

To validate femoral strength prediction, a rate-dependent material model, with different strain limit values for yield and failure, was used (Grassi et al. 2016). The material model and failure criterion are depicted in Fig. 2. The FE analyses were conducted in displacement control with consecutive 0.05 mm increments. The sum of the reaction forces at the increment where the first element of the model failed was calculated to indicate the predicted femoral strength. The simulation time was adjusted to provide a displacement rate of 15 mm/s, identical to the value used in the experimental mechanical tests. The predicted and experimental femoral strength data were compared in terms of relative error and standard error of the estimate (SEE). Again, the accuracy of the strength prediction achieved by CT-based FE models (Grassi et al. 2016) was presented to enable immediate comparison.

## Results

**Fig. 3:**
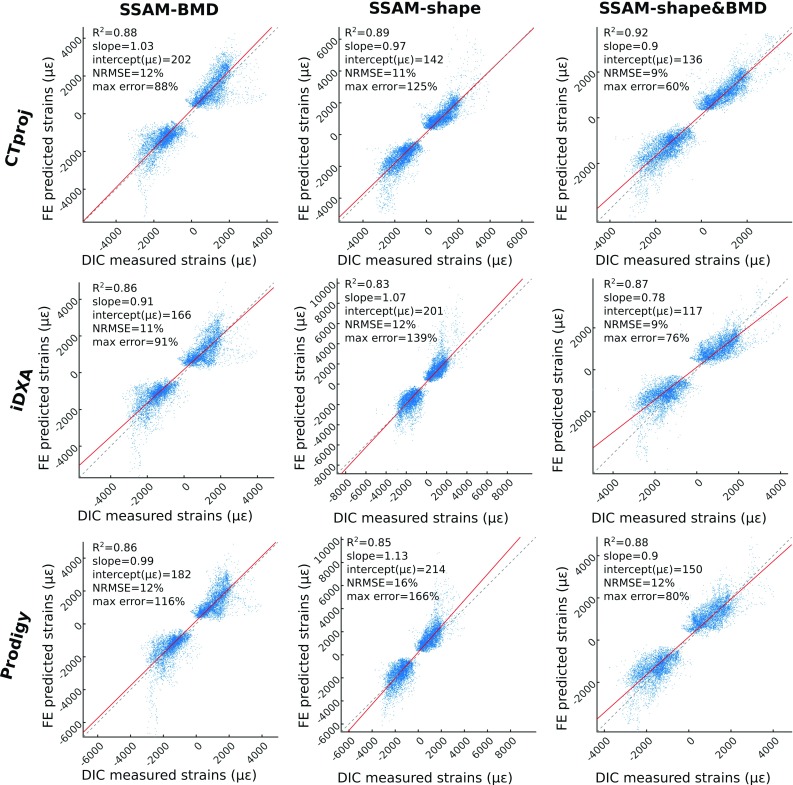
Prediction accuracy for the major and minor principal strains for *SSAM–BMD* (*first column*), *SSAM-shape* (*second column*), and *SSAM-shape* and *BMD* (*third column*) models of the three bones pooled together. From *top* to *bottom*, the accuracy results are plotted for the models using CT projection, iDXA, and Prodigy images for the SSAM-based reconstructions

The results of the robust regression analyses for the principal strains predicted at 4 BW are reported in Fig. 3 for the three bones pooled of the *SSAM–BMD*, *SSAM-shape*, and *SSAM-shape* and *BMD* models. The coefficient of determination ($${R}^{2})$$ was always greater than 0.83, while the slope was within ±10% from unity for all but two cases (SSAM-shape models from Prodigy images, and *SSAM-shape* and *BMD* models from iDXA images). The coefficient of determination was consistently higher for the models using the CT projection for the reconstruction, followed by those using Prodigy images. The models based on the use of iDXA images showed the lowest values. For comparison, the analogous robust regression analysis for the CT-based models (Grassi et al. 2016) when the data on three bones were pooled provided an $$R^{2}$$ of 0.94, with a slope of 0.96 $$(\hbox {intercept} = 133\,\upmu \upvarepsilon )$$, $$\hbox {NRMSE} = 9\%$$, with a maximum estimation error of 65%.

**Table 2:** Prediction accuracy for the major and minor principal strains for *SSAM–BMD* models of the three bones taken individually

	Bone #1			Bone #2			Bone #3		
	CTproj	iDXA	Prodigy	CTproj	iDXA	Prodigy	CTproj	iDXA	Prodigy
*SSAM–BMD*									
${R}^{2}$	0.9	0.9	0.9	0.84	0.83	0.83	0.92	0.89	0.89
Slope	1	0.91	0.92	1.03	0.99	1.08	1.03	0.85	0.97
Intercept (${{\upmu }{\upvarepsilon }}$)	225	199	200	257	263	283	142	84	107
NRMSE	13%	11%	11%	19%	18%	20%	12%	12%	12%
Max error%	64%	69%	70%	89%	89%	113%	63%	58%	80%
*SSAM-shape*									
${R}^{2}$	0.89	0.82	0.82	0.89	0.88	0.88	0.91	0.79	0.83
Slope	0.88	1	1.04	0.98	1.03	1.11	1.07	1.22	1.23
Intercept (${{\upmu }{\upvarepsilon }}$)	201	309	332	102	167	158	61	127	141
NRMSE	12%	18%	19%	13%	15%	18%	10%	13%	15%
Max error%	73%	188%	87%	125%	136%	108%	82%	134%	176%
*SSAM-shape and BMD*									
${R}^{2}$	0.88	0.84	0.86	0.94	0.89	0.9	0.92	0.87	0.88
Slope	0.81	0.76	0.88	0.9	0.74	0.86	0.98	0.86	0.99
Intercept (${{\upmu }{\upvarepsilon }}$)	197	217	252	109	141	181	68	3	17
NRMSE	11%	14%	15%	10%	11%	13%	9%	8%	12%
Max error%	34%	37%	43%	51%	70%	61%	74%	72%	91%
*CT-based* (Grassi et al. 2016)									
${R}^{2}$	0.92			0.94			0.95		
Slope	0.92			0.97			1.01		
Intercept (${{\upmu }{\upvarepsilon }}$)	144			174			79		
NRMSE	10%			11%			11%		
Max error%	46%			59%			83%		

For each bone, the accuracy obtained using the three different 2D reference images (CT projection, iDXA, and Prodigy) for the SSAM-based reconstruction is reported. The accuracy parameters reported by Grassi et al. (2016) for the CT-based models were also reported in the last row to allow for an easy comparison

The individual validation of the single bones demonstrated a coefficient of determination greater than 0.79 for all cases and a NRMSE always below 20%, as shown in Table 2 for the *SSAM–BMD*, *SSAM-shape*, and *SSAM-shape* & *BMD* models. The slope was generally close to unity, with a few exceptions: the slope was underestimated by 14–26% for the *SSAM-shape* and *BMD* models using iDXA images for the reconstruction. On the other hand, the slope of SSAM-shape models for bone #3 was overestimated by 22% and 23% when using iDXA and Prodigy images for reconstruction, respectively. The previous results of the analogous individual validations for the CT-based FE models in Grassi et al. (2016) are also reported in Table 2.

**Fig. 4:**
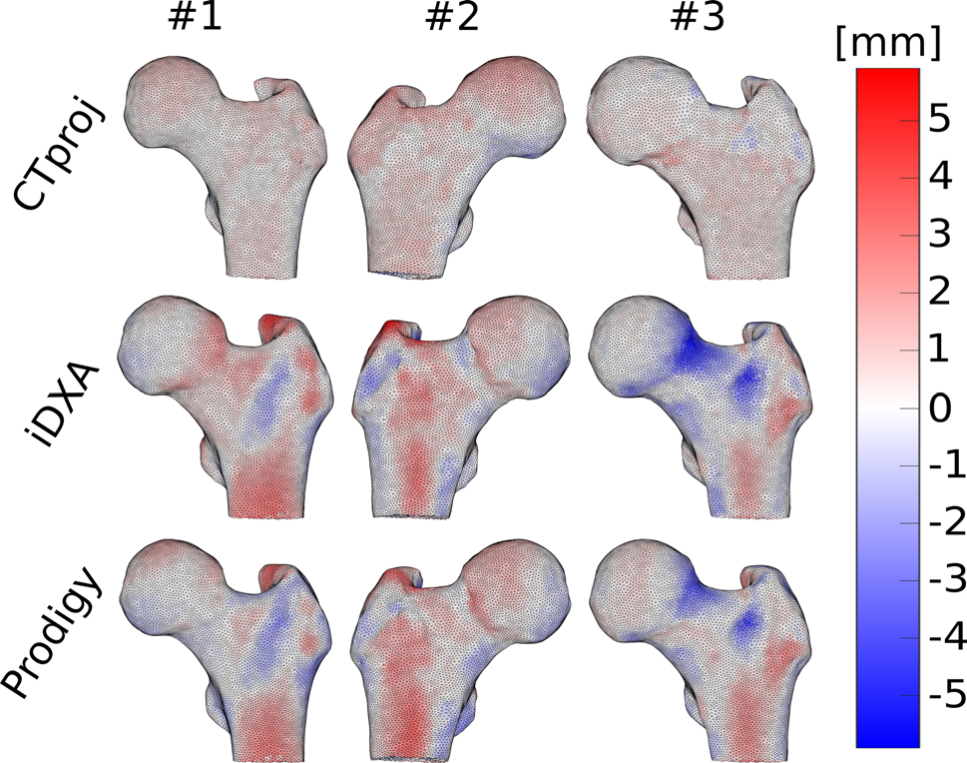
Error in the shape reconstruction for the three different femora (from *left* to *right*, bone #1, #2, and #3) and the different types of images (from *top* to *bottom*, CT projection, iDXA, and Prodigy) used for the SSAM-based reconstruction

**Table 3:** Relative change between the volume of the femoral neck of the *SSAM-shape* models and the CT-based models (here considered as the true value), for the three different types of 2D reference image (CT projection, iDXA, and Prodigy)

	Bone #1 (%)	Bone #2 (%)	Bone #3 (%)
CTproj	9	10	9
iDXA	19	13	$-$6
Prodigy	5	15	0.3

Positive values indicate that the SSAM-reconstructed shape is bigger

**Table 4:** Prediction accuracy for the major and minor principal strains in the femoral neck region only for *SSAM–BMD* models, for the three bones pooled and for each individual bone

	3 bones pooled			Bone #1			Bone #2			Bone #3		
	CTproj	iDXA	Prodigy	CTproj	iDXA	Prodigy	CTproj	iDXA	Prodigy	CTproj	iDXA	Prodigy
*SSAM–BMD*												
${R}^{2}$	0.81	0.77	0.76	0.83	0.83	0.83	0.76	0.73	0.74	0.88	0.83	0.81
Slope	0.84	0.76	0.83	0.90	0.87	0.84	0.77	0.77	0.91	0.86	0.69	0.80
Intercept (${{\upmu }{\upvarepsilon }}$)	103	80	94	245	249	235	$-$39	$-$42	$-$56	37	$-$19	$-$11
NRMSE	10%	11%	9%	17%	16%	15%	22%	25%	29%	14%	14%	17%
Max error%	67%	68%	71%	72%	74%	77%	98%	95%	115%	70%	66%	88%
*SSAM-shape*												
${R}^{2}$	0.86	0.82	0.84	0.90	0.82	0.83	0.87	0.89	0.90	0.87	0.77	0.81
Slope	0.96	1.16	1.16	0.88	0.98	0.97	1.08	1.15	1.16	0.97	1.37	1.34
Intercept (${{\upmu }{\upvarepsilon }}$)	97	268	261	197	353	366	$-$41	82	78	44	410	337
NRMSE	8%	8%	8%	12%	20%	18%	22%	18%	18%	12%	17%	19%
Max error%	74%	70%	74%	46%	64%	90%	125%	128%	88%	73%	128%	166%
*SSAM-shape and BMD*												
${R}^{2}$	0.89	0.85	0.86	0.88	0.83	0.83	0.93	0.87	0.89	0.88	0.84	0.86
Slope	0.89	0.78	0.91	0.79	0.71	0.81	0.95	0.75	0.88	0.95	0.93	1.08
Intercept (${{\upmu }{\upvarepsilon }}$)	163	110	154	269	245	291	27	27	67	60	51	84
NRMSE	10%	12%	12%	12%	14%	16%	13%	14%	16%	11%	10%	14%
Max error%	51%	65%	66%	41%	35%	39%	45%	66%	57%	71%	74%	91%
*CT-based* (Grassi et al. 2016)												
${R}^{2}$	0.91			0.89			0.93			0.93		
Slope	0.92			0.88			0.97			0.91		
Intercept (${{\upmu }{\upvarepsilon }}$)	124			182			23			100		
NRMSE	9%			13%			15%			11%		
Max error%	54%			40%			59%			49%		

The accuracy obtained using the three different 2D reference images (CT projection, iDXA, and Prodigy) for the SSAM-based reconstruction is reported. The accuracy parameters reported by the CT-based FE models presented in Grassi et al. (2016) for the femoral neck region only were also reported in the last row to allow for a direct comparison

The shape reconstructions performed over CTproj evidenced a higher accuracy in boundary recovery than those based on iDXA and Prodigy images (Fig. 4). Higher reconstruction errors were generally localized in regions with negligible contribution to the mechanical behaviour of femora, such as the tip of the greater trochanter. The volumetric difference at the femoral neck, calculated between the *SSAM-shape* and CT-based models (Table 3), highlighted the reconstruction error in a region with crucial mechanical contribution under the single leg stance configuration.

The results of the robust regression analyses performed considering only the femoral neck region are reported in Table 4 for the three bones pooled as well as for the individual bones. The coefficient of determination for the individual validations for the neck region was always greater than 0.73, with a NRMSE below 29%, with the *SSAM-shape* and *BMD* models providing $R^{2} > 0.85$ and NRMSE <12% for the three bones pooled.

**Table 5:** Femoral strength prediction accuracy for bones #1 and #2, for the three different FE models (*SSAM–BMD*, *SSAM-shape*, and *SSAM-shape* & *BMD*), each of them built for the three different 2D reference images (CT projection, iDXA, and Prodigy)

	Bone #1			Bone #2			SEE (N)
	CTproj	iDXA	Prodigy	CTproj	iDXA	Prodigy	
SSAM–BMD	9858 ($-$26%)	11,309 ($-$15%)	11,007 ($-$18%)	7115 ($-$9%)	7789 ($-$1%)	5046 ($-$35%)	2267
SSAM-shape	12,776 ($-$4%)	9301 ($-$30%)	10,983 ($-$18%)	7885 (+0.4%)	8525 (+8%)	7445 ($-$5%)	1975
SSAM-shape and BMD	13,106 ($-$2%)	13,009 ($-$3%)	14,820 (+11%)	9777 (+24%)	9203 (+17%)	8859 (+13%)	1215
CT-based (Grassi et al. 2016)	13,184 ($-$1%)			7947 (+1%)			155
Experimentally measured (Grassi et al. 2014b)	13,383			7856			–

The relative error to the actual femoral strength measured experimentally (Grassi et al. 2014b) is reported between parentheses. The strength prediction accuracy reported by Grassi et al. (2016) for the CT-based models was also reported in the last row to allow for an easy comparison

Due to a technical problem during the mechanical test, the femoral strength could not be validated for bone #3 (please see Grassi et al. 2016). The SEE for the *SSAM-shape* and *BMD* models (pooling the models from the three 2D reference images) was 1215 N (Table 5). Typically, more accurate strength estimations were obtained when using CTproj data as the 2D reference image (SEE $=$ 1689 N, against SEE equal to 1974 and 1938 N for iDXA and Prodigy data, respectively).

## Discussion

This study posed the main question of how accurately a 3D FE model reconstructed from a single DXA image and a SSAM could predict tissue strains and strength of proximal femur. The gold standard method, CT-based 3D FE model, was applied as a reference. To properly answer this question, it is necessary to understand the relative contribution of the different factors (bone shape, BMD distribution, local reconstruction errors, etc.) to the prediction accuracy. To this aim, three different models were built, implementing the reconstructed bone shape only (*SSAM-shape* models), the reconstructed BMD distribution only (*SSAM–BMD* models), and the combination of these two (*SSAM-shape* and *BMD* models).

The *SSAM-shape* and *BMD* models predicted strains with high accuracy (${R}^{2}> 0.87$, $\hbox {NRMSE} <12\%$ for the three bones pooled, Fig. 3, ${R}^{2} > 0.84$ and $\hbox {NRMSE} < 15\%$ for the individual bone validations, Table 2), when validated against thousands of experimental strain measurements per bone collected with DIC. CT-based FE models obtained a coefficient of determination of 0.94, with a NRMSE of 9% for the same set of samples and experimental data (Grassi et al. 2016). Our present results on accuracy were only slightly lower. Nevertheless, *SSAM-shape* and *BMD* models reconstructed using iDXA significantly underestimated the strain levels (slope of the robust linear regression $=$ 0.78). The inaccuracy was mostly related to bones #1 and #2 (*SSAM-shape* and *BMD* models from iDXA, Table 2). By implementing only the shape and only the BMD from the SSAM reconstruction, we can explain how the strain underestimation occurred for these two cases. When implementing only *SSAM–BMD* (*SSAM–BMD* models from iDXA, Table 2) and *SSAM-shape* (*SSAM-shape* models from iDXA, Table 2), both samples exhibited a slope close to unity. However, due to the shape reconstruction error, the volume in the femoral neck region was overestimated by 13–19% (iDXA values, Fig. 4; Table 3). Therefore, a correct reconstruction of the material properties was associated with a femoral neck that was 13–19% bigger in volume than the CT-based one, which led to a stiffer femoral neck and ultimately underestimated the principal strains for the iDXA cases. This reveals that the accuracy in the shape reconstruction from SSAM models should be evaluated not only in terms of the absolute point-to-surface distance, but also in terms of the capacity to preserve the actual volumes of the different anatomical compartments (femoral neck first, but also femoral head, and shaft).

The accuracy of the strain prediction decreased slightly when only the femoral neck region was considered ($R^{2} = 0.85{-}0.89$ for the *SSAM-shape* and *BMD* models of the three bones pooled, Table 4). This result could be expected, since femoral neck is a region where FE models typically exhibit a lower accuracy in predicting strains (Helgason et al. 2016). A decrease in strain prediction accuracy in the femoral neck region was observed for the CT-based models as well (${R}^{2} = 0.91$ for the three bones pooled, Table 4). When looking at the individual validations in the femoral neck region, the accuracy of the strain prediction for the SSAM-based models seems again to follow the accuracy of the point-to-surface reconstruction error (Fig. 4) and of the volumetric difference (Table 3). This trend further stresses the importance of an accurate reconstruction of both shape and BMD distribution in regions particularly prone to fracture, such as the femoral neck.

The *SSAM-shape* and *BMD* models predicted femoral strength with a SEE of 1215 N and a maximum absolute relative error of 24% (Table 5). The CT-based models predicted femoral strength with a SEE of 155 N and a maximum absolute relative error of 1.5% for the same set of data (Grassi et al. 2016). The accuracy data on femoral strength (Table 5) were scattered. Typically, femoral strength was predicted with high accuracy, but also some outliers with lower accuracy were found in the data. At least two main trends were observed, namely (1) the models built using CTproj as 2D reference image were more accurate than those built with iDXA and Prodigy. This was expected, since CTproj data had a higher signal-to-noise ratio than DXA images and represented an ideal reference image (although of no practical use, since a CT scan is needed). The comparison of the accuracy between CTproj and the two DXA images thus provides an estimation of the effect of image noise on the accuracy of the reconstruction. Interestingly, the different spatial resolution between iDXA (pixel size $0.25 \times 0.3$ mm) and Prodigy (pixel size $1.05 \times 0.6$ mm) was not found to affect the accuracy of the SSAM-based models. (2) The *SSAM–BMD* models had a higher SEE than the *SSAM-shape* models. This evidence suggested that the error in the reconstruction of BMD, and consequently of the material properties, influenced the outcome more than the error in the shape reconstruction. Consistent to this finding, Bonaretti et al. (2014) found that the mesh-based SSAM reconstructions (like the one used in this study) are less accurate than the image-based SSAM reconstructions in estimating the original bone density distribution. This was also consequent to the fact that a strain-based criterion, thus strongly dependent on the correctly estimated value for modulus of elasticity, was adopted for the calculation of femoral strength.

This is, to our best knowledge, the first study evaluating the ability of a FE model built from a statistical-based reconstruction to predict strains and femoral strength of human proximal femora anatomies against direct ex vivo measurements. A validation in terms of strain prediction accuracy was already proposed (Grassi et al. 2014a). However, the femoral strength was not evaluated and, more importantly, the PCA-based reconstruction was performed over the 3D CT data (Grassi et al. 2014a). This limited the applicability of the study to the reconstruction of synthetic anatomies aimed at exploring the effects of anatomical variability. In the present study, the FE models were reconstructed from two-dimensional reference images, thus making them suitable for subject-specific estimation of fracture risk. Earlier, Thevenot et al. (2014) validated their models in terms of femoral strength and reported a SEE of 543 N, a lower value than the SEE reported in the present study (SEE $=$ 1215 N, Table 5). However, the present samples were tested in a configuration resembling single leg stance. They were fractured at an average load of 10,620 N. Thevenot et al. tested their samples in an experimental configuration resembling a fall to the side, with a much lower fracture load [average 3188 N, as extrapolated by digitalization of data from Figure 4 in Thevenot et al. (2014)]. The present higher SEE is therefore consistent with the fracture load being three times higher than that found by Thevenot et al. In terms of relative error, the maximum absolute relative error in the prediction of femoral strength was 24% in our study, whereas it was 54% in Thevenot et al. [as extrapolated by digitalization of data from Figure 4 in Thevenot et al. (2014)].

Other studies have also proposed to use PCA-based models to predict fracture risk (Gregory et al. 2004; Schuler et al. 2010; Whitmarsh et al. 2012). However, those studies used the reconstructed shape and BMD distribution either to obtain three-dimensional anatomical and densitometry measurements that complemented the standard estimation of fracture risk, or to employ the model parameters as features for the classification. The present study, instead, used SSAM-based models to predict femoral strength using a purely mechanistic approach, analogously to how it is done with gold standard CT-based FE models.

The present study is limited by its small sample size, with three proximal human femora tested. However, the accuracy of the adopted SSAM-based method in reconstructing shape and BMD was previously validated using a higher number of samples (Väänänen et al. 2015). As the present focus was on the ability of the reconstructed models to predict strain and femoral strength, only the samples for which full-field strain data from ex vivo mechanical tests were available (Grassi et al. 2014b) were used. As another limitation, the adopted SSAM was trained on 34 femoral anatomies (Väänänen et al. 2015). Future works should aim at creating the SSAM using larger training sets, possibly also exploring the definition of different training sets for different population groups as defined by gender and ethnicity.

The combination of the current epidemiological-based estimation of individual fracture risk could be greatly improved by the addition of a mechanistic prediction of the load that a bone can bear without fracturing (Viceconti et al. 2015). When aiming to manage effectively the future challenges related to known increase of musculoskeletal diseases, such as osteoporosis and bone fractures, we are much limited with the existing medical technology. DXA is the current clinical standard to diagnose osteoporosis and ultimately estimate fracture risk. Adoption of CT for this screening is not realistic in a short-term scenario. Therefore, the current study aimed to improve the understanding of how useful the 3D FE models, as reconstructed from a single 2D DXA image, are to predict femoral strength. Based on the present findings, SSAM-based FE models provided a highly accurate representation of the subject-specific bone mechanics in terms of bone strains (${R}^{2} > 0.87$, $\hbox {NRMSE} < 12\%$). However, the accuracy in the prediction of femoral strength was inferior to those obtained with the state-of-the-art CT-based models (SEE $=$ 1215 N, against SEE $=$ 155 N for the CT-based models). The greater error in femoral strength estimation was mostly due to the presence of a few outliers in the data (Table 5). The present results highlight the potential of SSAM-based FE models to become a tool that provides a mechanistic prediction of fracture risk in a future clinical scenario. While an enlargement of both the population of validated specimens and the population of the SSAM training set is advocated before implementing the proposed SSAM-based approach in clinical trials, the present results could help to tailor future development of SSAM-based reconstructions with the aim to further improve their accuracy towards that of CT-based models.

## References

[CR1] Bayraktar HH, Morgan EF, Niebur GL (2004). Comparison of the elastic and yield properties of human femoral trabecular and cortical bone tissue. J Biomech.

[CR2] Bonaretti S, Seiler C, Boichon C (2014). Image-based vs. mesh-based statistical appearance models of the human femur: implications for finite element simulations. Med Eng Phys.

[CR3] Bryan R, Nair PB, Taylor M (2009). Use of a statistical model of the whole femur in a large scale, multi-model study of femoral neck fracture risk. J Biomech.

[CR4] Burge R, Dawson-Hughes B, Solomon DH (2007). Incidence and economic burden of osteoporosis-related fractures in the United States, 2005–2025. J Bone Miner Res.

[CR5] Castro-Mateos I, Pozo JM, Cootes TF (2014). Statistical shape and appearance models in osteoporosis. Curr Osteoporos Rep.

[CR6] Cody DD, Gross GJ, Hou FJ (1999). Femoral strength is better predicted by finite element models than QCT and DXA. J Biomech.

[CR7] Cummings SR, Cawthon PM, Ensrud KE (2006). BMD and risk of hip and nonvertebral fractures in older men: a prospective study and comparison with older women. J Bone Miner Res.

[CR8] Dall’Ara E, Eastell R, Viceconti M (2016). Experimental validation of DXA-based finite element models for prediction of femoral strength. J Mech Behav Biomed Mater.

[CR9] Engelke K, Lang T, Khosla S (2015). Clinical use of quantitative computed tomography (QCT) of the hip in the management of osteoporosis in adults: the 2015 ISCD official positions-Part I. J Clin Densitom.

[CR10] Grassi L, Schileo E, Boichon C (2014). Comprehensive evaluation of PCA-based finite element modelling of the human femur. Med Eng Phys.

[CR11] Grassi L, Väänänen SP, Amin Yavari S (2014). Full-field strain measurement during mechanical testing of the human femur at physiologically relevant strain rates. J Biomech Eng.

[CR12] Grassi L, Väänänen SP, Ristinmaa M (2016). How accurately can subject-specific finite element models predict strains and strength of human femora? Investigation using full-field measurements. J Biomech.

[CR13] Gregory JS, Testi D, Stewart A (2004). A method for assessment of the shape of the proximal femur and its relationship to osteoporotic hip fracture. Osteoporos Int.

[CR14] Helgason B, Gilchrist S, Ariza O (2016). The influence of the modulus-density relationship and the material mapping method on the simulated mechanical response of the proximal femur in side-ways fall loading configuration. Med Eng Phys.

[CR15] Järvinen TLN, Jokihaara J, Guy P (2014). Commentary conflicts at the heart of the FRAX tool. CMAJ.

[CR16] Järvinen TLN, Sievänen H, Jokihaara J, Einhorn TA (2005). Revival of bone strength: the bottom line. J Bone Miner Res.

[CR17] Johnell O, Kanis JA (2006). An estimate of the worldwide prevalence and disability associated with osteoporotic fractures. Osteoporos Int.

[CR18] Kanis JA (2002). Diagnosis of osteoporosis and assessment of fracture risk. Lancet.

[CR19] Kanis JA, Borgstrom F, De Laet C (2005). Assessment of fracture risk. Osteoporos Int.

[CR20] Kanis JA, McCloskey EV, Johansson H (2013). European guidance for the diagnosis and management of osteoporosis in postmenopausal women. Osteoporos Int.

[CR21] Langton CM, Pisharody S, Keyak JH (2009). Generation of a 3D proximal femur shape from a single projection 2D radiographic image. Osteoporos Int.

[CR22] Lekamwasam S (2010). Application of FRAX model to Sri Lankan postmenopausal women. J Clin Densitom.

[CR23] Liu A, Joe B (1994). Relationship between tetrahedron shape measures. BIT.

[CR24] MacNeil JAM, Adachi JD, Goltzman D (2012). Predicting fracture using 2D finite element modelling. Med Eng Phys.

[CR25] Morgan EF, Bayraktar HH, Keaveny TM (2003). Trabecular bone modulus-density relationships depend on anatomic site. J Biomech.

[CR26] Op Den Buijs J, Dragomir-Daescu D (2010). Validated finite element models of the proximal femur using two-dimensional projected geometry and bone density. Comput Methods Programs Biomed.

[CR27] Rho JY, Ashman RB, Turner CH (1993). Young’s modulus of trabecular and cortical bone material: ultrasonic and microtensile measurements. J Biomech.

[CR28] Sarkalkan N, Waarsing JH, Bos PK (2014). Statistical shape and appearance models for fast and automated estimation of proximal femur fracture load using 2D finite element models. J Biomech.

[CR29] Sarkalkan N, Weinans H, Zadpoor AA (2014). Statistical shape and appearance models of bones. Bone.

[CR30] Schileo E, Dall’ara E, Taddei F (2008). An accurate estimation of bone density improves the accuracy of subject-specific finite element models. J Biomech.

[CR31] Schuler B, Fritscher KD, Kuhn V (2010). Assessment of the individual fracture risk of the proximal femur by using statistical appearance models. Med Phys.

[CR32] Silverman SL, Calderon AD (2010). The utility and limitations of FRAX: a US perspective. Curr Osteoporos Rep.

[CR33] Taddei F, Schileo E, Helgason B (2007). The material mapping strategy influences the accuracy of CT-based finite element models of bones: an evaluation against experimental measurements. Med Eng Phys.

[CR34] Thevenot J, Koivumäki J, Kuhn V (2014). A novel methodology for generating 3D finite element models of the hip from 2D radiographs. J Biomech.

[CR35] Väänänen SP, Grassi L, Flivik G (2015). Generation of 3D shape, density, cortical thickness and finite element mesh of proximal femur from a DXA image. Med Image Anal.

[CR36] Venäläinen MS, Mononen ME, Väänänen SP (2016). Effect of bone inhomogeneity on tibiofemoral contact mechanics during physiological loading. J Biomech.

[CR37] Viceconti M, Hunter P, Hose D (2015). Big data, big knowledge: big data for personalised healthcare. IEEE J Biomed Heal Inform.

[CR38] Watts NB, Ettinger B, LeBoff MS (2009). FRAX facts. J Bone Miner Res.

[CR39] Whitmarsh T, Fritscher KD, Humbert L (2012). Hip fracture discrimination from dual-energy X-ray absorptiometry by statistical model registration. Bone.

[CR40] Whitmarsh T, Humbert L, De Craene M (2011). Reconstructing the 3D shape and bone mineral density distribution of the proximal femur from dual-energy X-ray absorptiometry. IEEE Trans Med Imaging.

[CR41] Yang L, Palermo L, Black DM, Eastell R (2014). Prediction of incident hip fracture with the estimated femoral strength by finite element analysis of DXA Scans in the study of osteoporotic fractures. J Bone Miner Res.

